# Genome-Wide Analysis of bZIP Transcription Factors and Expression Patterns in Response to Salt and Drought Stress in *Vaccinium corymbosum*

**DOI:** 10.3390/ijms26020843

**Published:** 2025-01-20

**Authors:** Xinghua Feng, Chuchu Wang, Sijin Jia, Jiaying Wang, Lianxia Zhou, Yan Song, Qingxun Guo, Chunyu Zhang

**Affiliations:** Department of Horticulture, College of Plant Science, Jilin University, Changchun 130062, China

**Keywords:** VcbZIPs, blueberry, salt stress, drought stress, ABA, RNA-seq

## Abstract

The basic leucine zipper (bZIP) transcription factors play essential roles in multiple stress responses and have been identified and functionally characterized in many plant species. However, the bZIP family members in blueberry are unclear. In this study, we identified 102 *VcbZIP* genes in *Vaccinium corymbosum*. *VcbZIPs* were divided into 10 groups based on phylogenetic analysis, and each group shared similar motifs, domains, and gene structures. Predictions of cis-regulatory elements in the upstream sequences of *VcbZIP* genes indicated that VcbZIP proteins are likely involved in phytohormone signaling pathways and abiotic stress responses. Analyses of RNA deep sequencing data showed that 18, 13, and 7 *VcbZIP* genes were differentially expressed in response to salt, drought, and ABA stress, respectively, for the blueberry cultivar Northland. Ten *VcbZIP* genes responded to both salt and drought stress, indicating that salt and drought have unique and overlapping signals. Of these genes, *VcbZIP1–3* are responsive to salt, drought, and abscisic acid treatments, and their encoded proteins may integrate salt, drought, and ABA signaling. Furthermore, *VcbZIP1–3* from group A and *VcbZIP83–84* and *VcbZIP75* from group S exhibited high or low expression under salt or drought stress and might be important regulators for improving drought or salt tolerance. Pearson correlation analyses revealed that VcbZIP transcription factors may regulate stress-responsive genes to improve drought or salt tolerance in a functionally redundant manner. Our study provides a useful reference for functional analyses of VcbZIP genes and for improving salt and drought stress tolerance in blueberry.

## 1. Introduction

The basic leucine zipper (bZIP) transcription factor (TF) family is widely distributed in eukaryotes. These transcription factors regulate gene expression by interacting with the promoter regions of downstream genes [1,2]. The bZIP proteins encoded by the bZIP gene family contain the highly conserved bZIP domain (60–80 amino acids), which is characterized by a basic DNA-binding domain and an adjacent leucine zipper, enabling bZIP to dimerize [3,4]. The bZIP protein usually forms dimers through the leucine zipper [5,6]. bZIP monomers form homodimers and heterodimers in different environments. The diversity of dimers results in enormous regulatory flexibility and expands the number and ability of bZIP dimers to bind target genes [7].

bZIP TF families have been identified in multiple model plants and many horticulture plants such as grapevine (*Vitis vinifera*), apple (*Malus domestica*), peach (*Prunus persica*), strawberry (*Fragaria vesca*), and banana (*Musa acuminata*) [8,9,10,11]. In Arabidopsis, bZIP genes were assorted into 13 groups (A-K, M, and S) involved in multiple functions related to plant development, environmental signaling, and stress response. Abscisic acid (ABA)-responsive element binding factors (ABF1–4) were clustered in group A and act at the core of ABA signaling in a partially redundant manner [1,12]. In general, ABFs are induced by various abiotic stresses, including drought, salinity, cold, and the exogenous hormone ABA, and control the expression of their stress-responsive target genes by directly binding to ABA-responsive elements [13,14]. AtbZIP17 (group B) is a transcriptional activator involved in salt and stress response, AtbZIP1 (group S) transcription factor is a positive regulator of plant tolerance to salt and drought stresses, and AtbZIP24 (group F) was proposed to act as a negative regulator in salt-stress response [15,16,17,18,19]. Thus, the bZIP genes of different groups are involved in abiotic stress response and perform various functions.

Plants are frequently exposed to various abiotic stresses such as drought, heat, cold, salt, and nutrient deficiency during their life cycles. Salt and drought are major environmental factors that limit plant productivity and threaten food security [20,21]. The increasing frequency of extreme weather exacerbates the adverse effects of salt and drought stress on plant growth and development [22]. Both salt and drought stress cause osmotic stress, resulting in reactive oxygen species (ROS) accumulation. When ROS are overgenerated in cells, they damage DNA, proteins, membranes, and lipids and inhibit plant growth. To regulate oxidative stress and keep ROS at a basic nontoxic level, eukaryotic cells produce different ROS-scavenging enzymes, such as superoxide dismutase (SOD), catalase isozyme (CAT), and glutathione S-transferase (GST), to improve plant tolerance to salt and drought stress. The transcription factors, such as bZIP, MYB, bHLH, WRKY, and ethylene response factor (ERF), receive stress signals and transmit the signals to activate the expression of downstream genes, imparting plant tolerance to abiotic stresses [12,23,24,25]. For example, overexpression of *Malus xiaojinensis MxWRKY64* or *MxWRKY53* genes in Arabidopsis increased the activities of SOD, POD, and CAT and enhanced the tolerance to salt stress, and overexpression of *Malus baccata MbWRKY1* and *MbWRKY4* genes in tobaccos enhanced the expressions of oxidative stress response genes (*NtPOD*, *NtCAT*, *NtSOD*, and *NtAPX*) and improved drought stress tolerance in transgenic plants [26,27,28,29]. However, the molecular mechanism of plant response to abiotic stress is multi-level and multi-process and is a complex response mechanism with multiple genes, signaling pathways, and metabolic processes [30]. Thus, salt and drought have unique and overlapping signals. The ABA signaling pathway is central to salt and drought stress responses in plants [31]. An important feature of salt and drought stress is that the hyperosmotic signal causes the accumulation of the phytohormone ABA. In this signaling pathway, the bZIP-type AREB/ABF/ABI5 transcript factor regulates the expression of downstream target genes to enhance tolerance to salt and drought stress [32,33].

Blueberries (*Vaccinium* sp.) are native to North America and have sharply increased in popularity worldwide in recent years due to their known nutritional and economic value and health benefits. The rising demand for blueberry products has led to the global expansion of blueberry cultivation [34,35]. However, blueberries are particularly vulnerable to adverse soil conditions due to their superficial root system and lack of root hairs. The roots of blueberry plants grow to less than 40 cm deep, which limits their ability to absorb water and nutrients from the soil and limits production [36,37]. In China, the main distribution of commercial blueberries from north to south comprises lowbush blueberry (*V. angustifolium*), highbush blueberry (*V. corymbosum*), and rabbiteye blueberry (*V. ashei*), of which highbush blueberry is the most important species. The *Vaccinium corymbosum/angustifolium* cultivar Northland is a half-highbush blueberry with strong cold tolerance, high yield, and good taste and is the main cultivar in Jilin Province, China [38]. Therefore, it is important to explore the function of bZIP TFs of blueberry under salt and drought stress and breed salt-tolerant or drought-resistant blueberry varieties for the development of the blueberry industry in Jilin Province, China. Although high-quality genomes of blueberry have been released, those of half-highbush blueberry have not yet been reported. The *V. corymbosum* cv. Draper genome database offers the possibility to systematically identify and investigate the putative functions of gene family members in blueberry because of its high-quality data [38,39,40].

In this study, we identified 102 *VcbZIP* genes from the Genome Database for Vaccinium and conducted comprehensive analysis, including physicochemical properties, evolutionary relationships, gene structures, protein motifs, and the cis-regulatory elements in the promoters. We then analyzed the responses of the *VcbZIP* genes to salt, drought, and ABA stress based on transcriptome deep sequencing (RNA-seq) from the cultivar Northland. In addition, we examined the correlations between *VcbZIP* and stress-responsive genes under salt and drought stress. Finally, the function of VcbZIPs was analyzed to preliminarily reveal their role in salt and drought stress. Our results provide a new basis for the future functional characterization of blueberry cultivar Northland *VcbZIP* genes and provide candidate genes for use in breeding for blueberry salt and drought tolerance.

## 2. Results

### 2.1. Identification and Physicochemical Properties of VcbZIP Family Members in Blueberry

To identify putative *VcbZIP* genes in the blueberry genome, an HMM search was conducted with bZIP domains (PF00170, PF07716, PF03131, and PF12498) and BLASTP using Arabidopsis bZIP genes as queries to search against the GDV. A total of 102 putative *VcbZIP* genes were identified after removing short and redundant sequences and named *VcbZIP1* through to *VcbZIP102* according to evolutionary analysis. The physicochemical properties and basic characteristics of the predicted 102 VcbZIPs were analyzed and are given in Table S1. The lengths of these VcbZIP proteins ranged from 109 (VcbZIP74) to 780 (VcbZIP31) amino acids, with the aliphatic index ranging from 48.69 (VcbZIP37) to 94.44 (VcbZIP73). The instability indices ranged from 30.27 to 77.88, and only those of VcbZIP95, VcbZIP97, VcbZIP98, and VcbZIP101 were less than 40, indicating that most VcbZIP proteins are unstable. All VcbZIPs have a negative grand average of hydropathy values, which indicates that all the VcbZIP proteins are hydrophilic proteins. The subcellular localization prediction indicated that all the VcbZIP proteins are located in the nucleus.

### 2.2. Phylogenetic Analysis of VcbZIP Proteins

**Figure 1:**
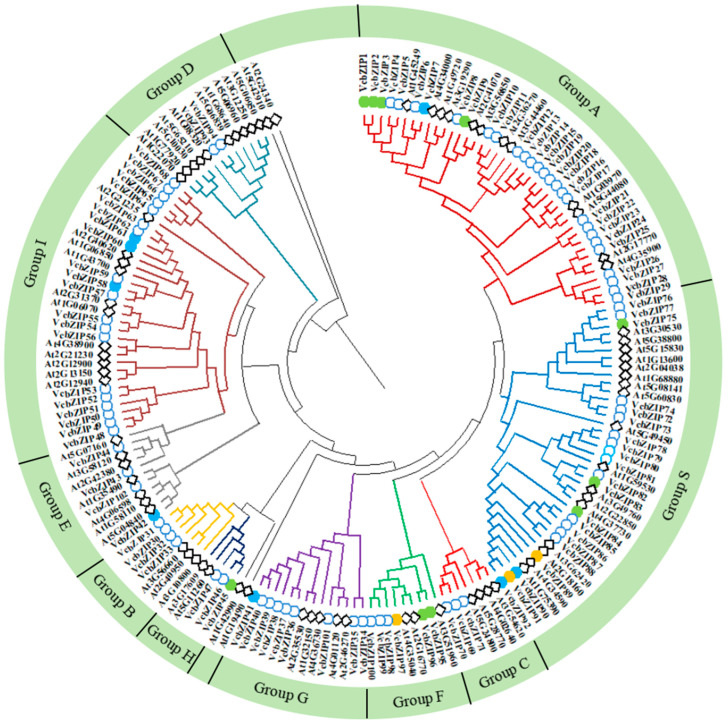
Phylogenetic analysis of bZIPs in blueberry (*Vaccinium corymbosum*) and Arabidopsis (*Arabidopsis thaliana*). Different colors represent the different groups. The blue circles and black diamonds represent the bZIP proteins from blueberry and Arabidopsis, respectively. Green, blue, and orange dots represent blueberry VcbZIP genes induced by salt and drought stress, salt stress, and drought stress, respectively.

We constructed a phylogenetic tree based on the amino acid sequences of the 102 blueberry VcbZIPs and 127 Arabidopsis AtbZIPs (Figure 1) to investigate the homologous evolutionary relationships and classification of VcbZIP family members. The VcbZIP proteins were divided into 10 groups (A–I and S) with reference to the evolutionary relationship and naming rules of the Arabidopsis bZIP proteins. In the VcbZIP family, the largest group is group A (29 members), followed by groups S and I (each with 21 members), and the smallest group is group D (2 members). The lengths of the VcbZIP proteins were small in group S (most genes had less than 200 amino acids) and large in group B (more than 700 amino acids). Kyoto Encyclopedia of Genes and Genomes (KEGG) pathway analysis showed that VcbZIP1–bZIP29 proteins (group A) were involved in ABA signaling, and VcbZIP45, VcbZIP46, and VcbZIP47 (group H) belonged to the elongated hypocotyl 5 (HY5) family (Table S1). All the VcbZIP proteins were distributed in 10 groups of Arabidopsis, suggesting that the bZIP gene family was conserved in evolutionary history.

### 2.3. Gene Structures of VcbZIP Proteins

**Figure 2:**
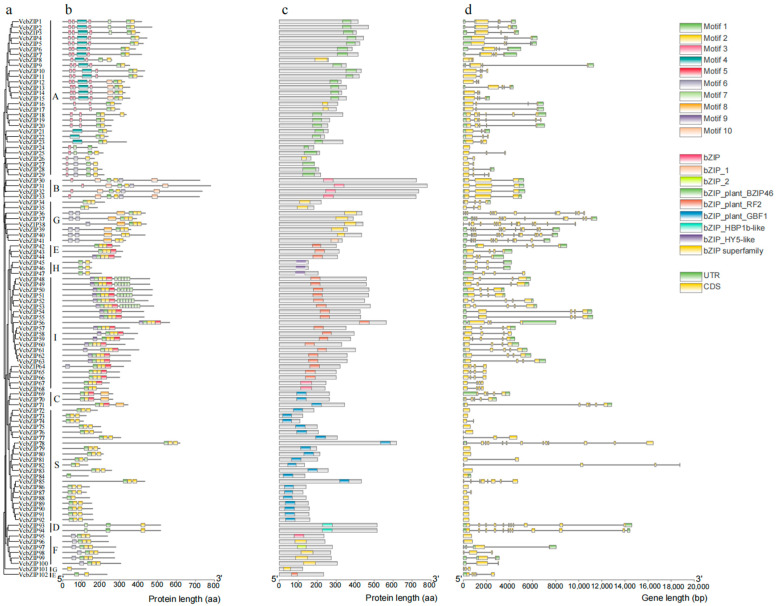
Diagrams of the motif compositions, conserved domains, and gene structures of VcbZIPs. (**a**) Phylogenetic tree of VcbZIPs. A total of 102 VcbZIPs were clustered in groups A–I and S. Different colors represent the different groups. (**b**) Conserved motif compositions of VcbZIPs. The maximum number of motifs was set to 10. Different colored boxes represent the corresponding conserved motifs on the upper right. (**c**) The conserved domains of VcbZIPs. Different colored boxes represent the corresponding conserved domains (right center). (**d**) Structures of the *VcbZIPs*. Dark green boxes, orange boxes, and gray lines represent UTRs, exons, and introns, respectively.

To gain further insight into the structural characteristics of *VcbZIP* genes, a rootless phylogenetic tree was constructed (Figure 2a), the conserved motifs were predicted (Figure 2b), the domains were identified (Figure 2c), and the exon–intron structures were analyzed (Figure 2d) for *VcbZIP* genes. We predicted the presence of 10 conserved motifs in these genes using the Multiple Em for Motif Elicitation (MEME) website, as shown in Figure S1, where the letter height of the amino acid residue represents its conservation degree. These motifs were distributed across different VcbZIPs, and the existence of conserved motifs in the same phylogenetic group of VcbZIPs was similar (Figure 2b). For example, most members of group A contained conserved motifs 1, 2, 3, and 4, while most members of group S conserved motifs 1 and 2. However, some groups had unique motifs. For example, group A had a unique motif 4, and a conserved motif 7 was only observed in group I. Furthermore, conserved motifs 1 and 2 were present in all groups; therefore, these two conserved motifs may be the main motifs in the VcbZIP family.

Conserved domain analysis reveals that the existence of domains in the same phylogenetic group of VcbZIPs was often highly conservative (Figure 2c). Most members of group A contained the bZIP_pant_BZIP46 domain; the bZIP_plant_RF2 domain was found in groups E and I; the bZIP_plant_GBF1 domain was shared by all the members of groups C and S; the bZIP_HBP1b-like domain was only owned by members of group D; all the members of group H contained the conserved bZIP_HY5-like domain; and the bZIP, bZIP_1, bZIP_2, and bZIP superfamily domains were found in groups A, B, G, and F. These results indicate that most VcbZIP members from the same group possess the same bZIP domains.

The exon–intron structures of *VcbZIP* genes were also characterized to better understand the structural evolution of the VcbZIP family (Figure 2d). We identified 15 *VcbZIP* genes with no introns, all of which belonged to groups A, S, and F and accounted for 15% of the total number of *VcbZIP* genes. The numbers of introns present in the VcbZIPs ranged from 0 to 13, and the same group showed similar numbers of introns. Most *VcbZIP* genes in group S contained 0 introns, group I contained 3–4 introns, group A contained 0–5 introns, group C contained 4–5 introns, group D contained 10–11 introns, and group G contained 10–12 introns. Overall, *VcbZIP* gene structures displayed diversity, and *VcbZIP* genes with close phylogenetic relationships may possess similar gene structures, among which the gene structures of group G were the most complex.

### 2.4. Analysis of Phytohormone- and Abiotic-Stress-Related Cis-Acting Elements in the VcbZIP Promoters

**Figure 3:**
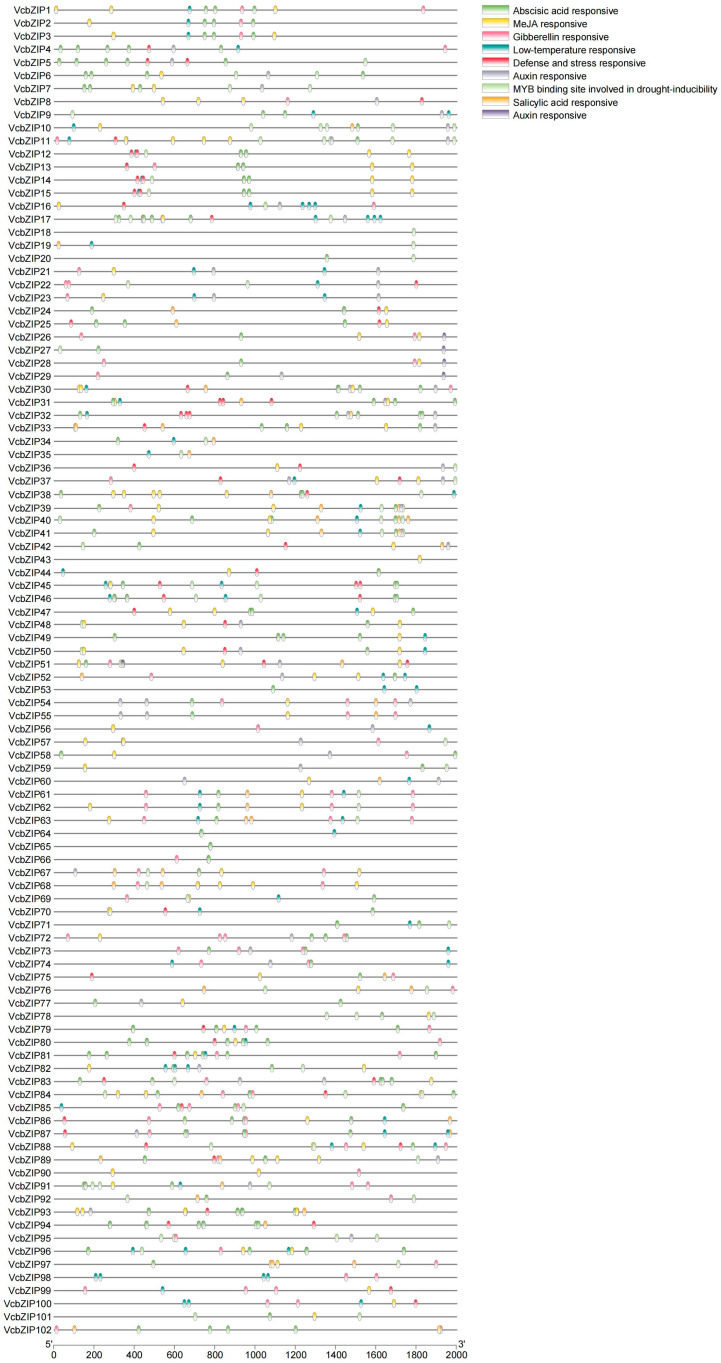
Diagram of the predicted cis-regulatory elements in the *VcbZIP* promoters. Different colored symbols represent cis-regulatory elements, as shown to the right of the diagram.

To explore the potential functions of VcbZIPs, the 2000 bp upstream promoter sequences from the ATG start codon of *VcbZIPs* were used to search for the cis-regulatory elements involved in phytohormone and abiotic stress using the PlantCARE online tool (Figure 3; Table S2). The *VcbZIP* promoter regions contained various cis-acting elements related to phytohormones, including abscisic acid, auxin, gibberellin, methyl jasmonate (MeJA), and salicylic acid-responsive elements, which was consistent with the function of bZIPs in response to abiotic stress. These promoters also contained many abiotic-stress-related elements, such as defense and stress-responsive elements, low temperature-responsive elements, and the MYB binding site involved in drought inducibility elements. Abscisic acid-responsive elements were present in almost all the *VcbZIP* promoter regions (82/102). We also found that 77% (79/102) of *VcbZIP* promoters contained MeJA-responsive elements, 56% (57/102) of *VcbZIP* promoters contained gibberellin-responsive elements, and 48% (49/102) of *VcbZIP* promoters contained auxin-responsive elements; however, only 35% (36/102) of *VcbZIP* promoters contained salicylic acid-responsive elements. In addition, 57 of 102 *VcbZIP* promoters contained low temperature-responsive elements, 54 of 102 *VcbZIP* promoters contained the MYB binding site involved in drought inducibility elements, and 44 of 102 *VcbZIP* promoters contained defense and stress-responsive elements (Table S2). These results indicate that *VcbZIP* genes may play important roles in plant response to abiotic stress.

### 2.5. The VcbZIP Expression Pattern in Response to Salt and Drought Stress

**Figure 4:**
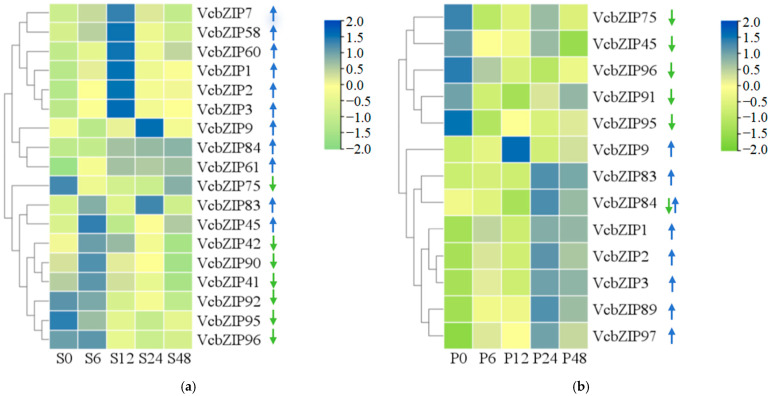
The heatmap of differentially expressed *VcbZIP* genes in response to salt (**a**) and drought (**b**) stress in blueberry leaves based on log_10_ (FPKM) values according to transcriptome deep sequencing (RNA-seq). Colored bars on the right indicate low expression (green) or high expression (blue) of differentially expressed genes. Blue upward and green downward arrows indicate that *VcbZIPs* are upregulated and downregulated by salt or drought stress, respectively.

We downloaded RNA-seq data from blueberry leaves under NaCl and PEG 6000 treatment conditions from the BioProject database (Tables S3 and S4) to study the expression patterns of *VcbZIPs* in response to salt and drought stress. Eighteen VcbZIPs were differentially expressed in response to salt stress, of which *VcbZIP1*, *VcbZIP2*, *VcbZIP3*, *VcbZIP7*, *VcbZIP9*, *VcbZIP45*, *VcbZIP61*, *VcbZIP83*, *VcbZIP84*, *VcbZIP58*, and *VcbZIP60* were upregulated, and *VcbZIP41*, *VcbZIP42*, *VcbZIP75*, *VcbZIP90*, *VcbZIP92*, *VcbZIP95*, and *VcbZIP96* were downregulated compared to the 0 h control samples. The expression levels of *VcbZIP1*, *VcbZIP2*, *VcbZIP3*, *VcbZIP7*, and *VcbZIP84* were highest at 12 h of salt stress (Figure 4a; Table S3). A total of 13 *VcbZIP* genes were differentially expressed in response to drought stress, of which *VcbZIP1*, *VcbZIP2*, *VcbZIP3*, *VcbZIP9*, *VcbZIP83*, *VcbZIP89*, and *VcbZIP97* were upregulated; *VcbZIP45*, *VcbZIP75*, *VcbZIP91*, *VcbZIP95*, and *VcbZIP96* were downregulated; and *VcbZIP84* was downregulated and then upregulated compared to the 0 h control samples. The expression levels of *VcbZIP1*, *VcbZIP2*, *VcbZIP3*, and *VcbZIP83* were highest at 24 h of drought stress (Figure 4b; Table S4). Both salt and drought significantly decreased the expression of *VcbZIP75* (Figure 4).

The RNA-seq data also showed that ten VcbZIP genes from group A (*VcbZIP1*, *VcbZIP2*, *VcbZIP3*, and *VcbZIP9*), group S (*VcbZIP75*, *VcbZIP83*, and *VcbZIP84*), group H (*VcbZIP45*), and group F (*VcbZIP95* and *VcbZIP96*) were expressed in response to both salt and drought stress (Figure 1). Of these genes, the expression of *VcbZIP1*, *VcbZIP2*, *VcbZIP3*, *VcbZIP9*, *VcbZIP83*, and *VcbZIP84* was upregulated, and that of *VcbZIP75*, *VcbZIP95*, and *VcbZIP96* was downregulated compared to the 0 h control samples under salt and drought stress. However, *VcbZIP45* was upregulated under salt stress and downregulated under drought stress (Figure 4a,b). These results indicated that most *VcbZIP* genes may have similar functions in response to salt and drought stress; however, some *VcbZIP* genes may play different roles under salt and drought stress.

Furthermore, the differentially expressed *VcbZIP* genes were identified as being expressed in response to ABA stress using RNA-seq data. However, only *VcbZIP1–7* from group A were induced and upregulated by 100 μM exogenous ABA stress. Thus, these seven *VcbZIP* genes may be involved in the ABA signaling pathways (Table S5).

**Figure 5:**
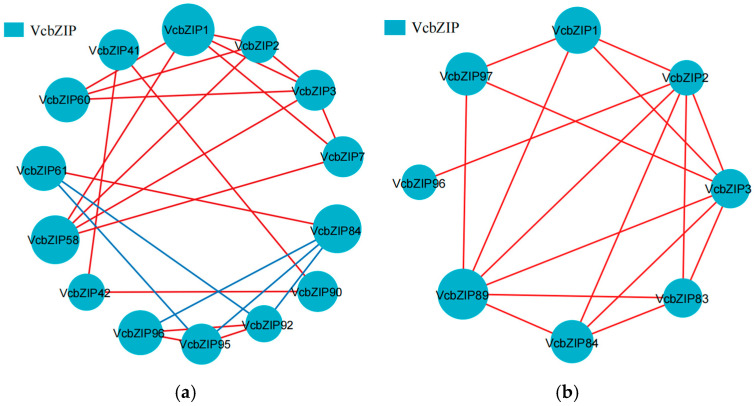
The network of Pearson correlation between *VcbZIP* genes under salt (**a**) and drought (**b**) stress. Red lines indicate positive correlations; blue lines indicate negative correlations. The size of the circle represents the number of related genes.

To investigate the roles of VcbZIPs in response to salt and drought stress, the correlation between the *VcbZIP* genes was analyzed using Pearson correlation coefficients during salt or drought stress (Tables S6 and S7). *VcbZIP1*, *VcbZIP2*, and *VcbZIP3* were positively correlated with regard to salt stress, and *VcbZIP7* was also positively correlated with *VcbZIP1* and *VcbZIP3* in expression levels. *VcbZIP1*, *VcbZIP2*, *VcbZIP3*, and *VcbZIP7* (group A) were positively correlated with *VcbZIP58* or *VcbZIP60* (group I). In group S, *VcbZIP84* was positively correlated with *VcbZIP61* (group I) and negatively correlated with *VcbZIP92*, *VcbZIP95*, and *VcbZIP96* (group S); *VcbZIP90* was positively correlated with *VcbZIP41* (group G) and *VcbZIP42* (group E); and *VcbZIP92* was positively correlated with *VcbZIP95* and *VcbZIP96* (group F) and negatively correlated with *VcbZIP61* (group I) during salt stress (Figure 5a). There was also a significant correlation between *VcbZIP1*, *VcbZIP2*, and *VcbZIP3* in the case of drought, and these three *VcbZIP* genes were positively correlated with *VcbZIP83*, *VcbZIP84*, or *VcbZIP89* in group S and *VcbZIP97* in group F. There was also a significant correlation between *VcbZIP83*, *VcbZIP84*, and *VcbZIP89* in group S during salt stress (Figure 5b).

**Figure 6:**
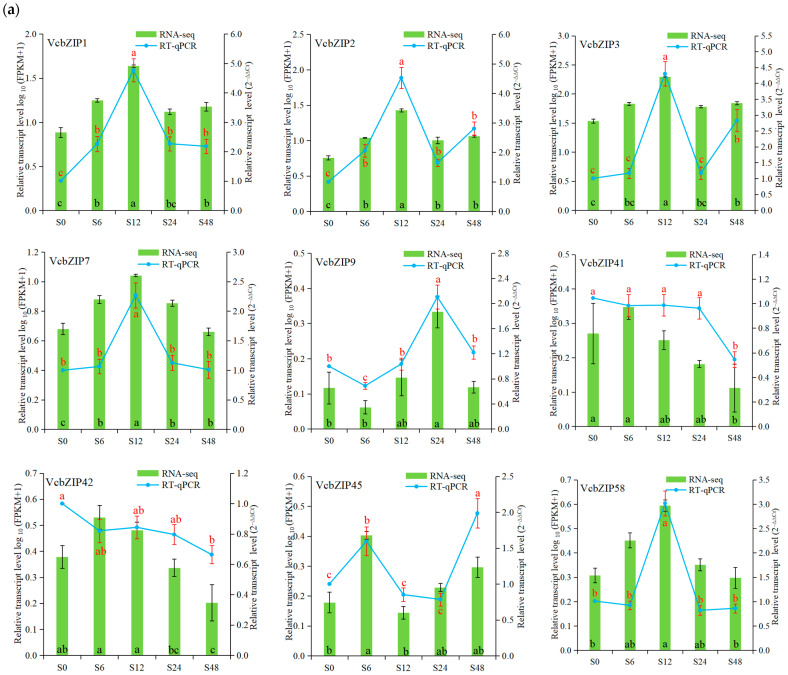

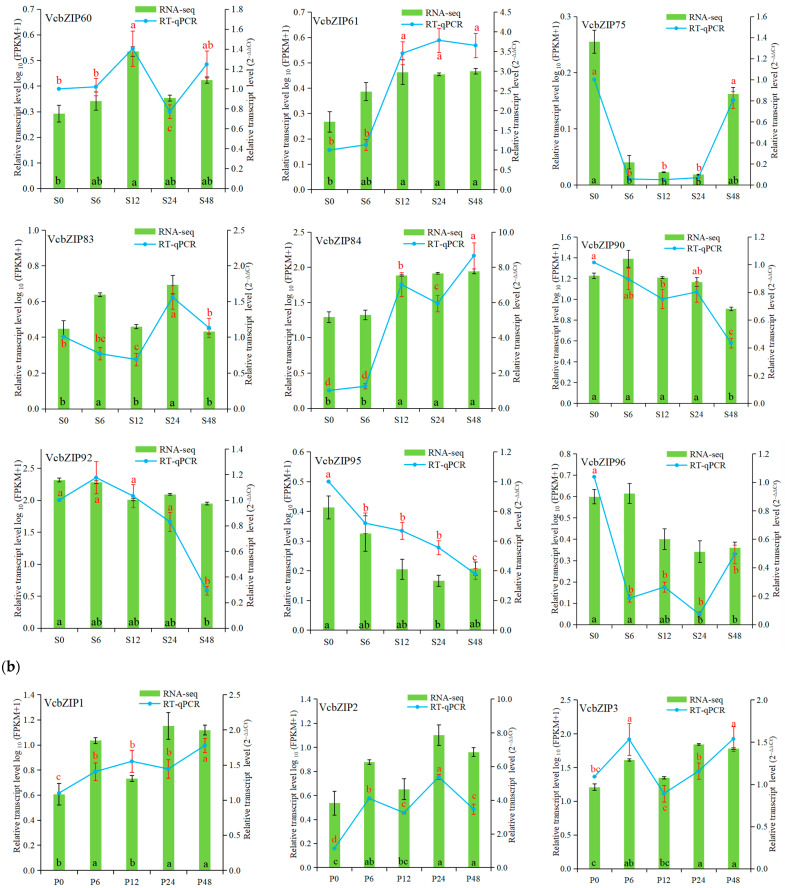

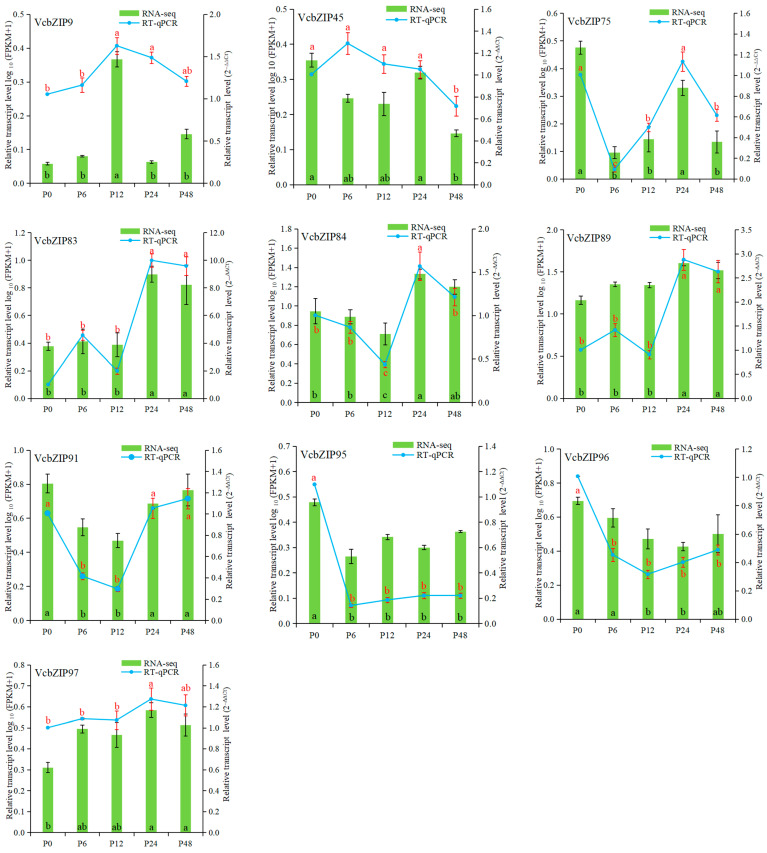
Expression patterns of *VcbZIPs* under salt (**a**) and drought (**b**) stress based on log_10_ (FPKM+1) values of RNA-seq data and based on 2^−∆∆Ct^ values of RT-qPCR data. Values are means ± SD from three independent biological replicates. Statistically significant differences were determined using Tukey’s test at a *p* value of ≤0.05. The different black and red letters indicate significant differences compared with the 0 h control for RNA-seq and RT-qPCR, respectively.

We examined the expression patterns of these differentially expressed *VcbZIP* genes using RT-qPCR to confirm the accuracy and reliability of the expression patterns based on RNA-seq data. Consistent with RNA-Seq data, the expression of *VcbZIP1*, *VcbZIP2*, *VcbZIP3*, *VcbZIP7*, *VcbZIP9*, *VcbZIP45*, *VcbZIP58*, *VcbZIP60*, *VcbZIP61*, *VcbZIP83*, and *VcbZIP84* was significantly upregulated, and that of *VcbZIP41*, *VcbZIP42*, *VcbZIP75*, *VcbZIP90*, *VcbZIP92*, *VcbZIP95*, and *VcbZIP96* was significantly downregulated under salt stress compared to the 0 h control samples (Figure 6a). The expression of *VcbZIP1*, *VcbZIP2*, *VcbZIP3*, *VcbZIP9*, *VcbZIP83*, *VcbZIP89*, and *VcbZIP97* was significantly upregulated, and that of *VcbZIP45*, *VcbZIP75*, *VcbZIP91*, *VcbZIP95*, and *VcbZIP96* was significantly downregulated under drought stress compared to the 0 h control samples. We also found that the expression level of the *VcbZIP84* gene was downregulated at 12 h and upregulated at 24 h under drought stress (Figure 6b). Furthermore, RT-qPCR data showed expression patterns similar to those of RNA-seq data for the same *VcbZIP* genes during salt or drought stress (Figure 6a,b). For example, both RNA-seq and RT-qPCR data showed that the expression levels of VcbZIP1 increased and reached the highest level at 12 h and then decreased during salt stress. Both RNA-seq and RT-qPCR data showed that the expression levels of *VcbZIP1*, *VcbZIP2*, *VcbZIP3*, *VcbZIP83*, and *VcbZIP84* significantly increased by more than 4 times under salt or drought stress compared to the 0 h control, and that of *VcbZIP75* significantly decreased by more than 8 times under salt and drought stress compared to the 0 h control. The expression levels of *VcbZIP84* and VcbZIP83 also significantly increased by more than 4 times under salt stress and drought stress, respectively, according to RNA-seq and RT-qPCR data. Thus, *VcbZIP1*, *VcbZIP2*, *VcbZIP3*, *VcbZIP83*, *VcbZIP84*, and VcbZIP75 may play important roles under salt and drought stress.

### 2.6. The Correlations Between VcbZIP Genes and Stress-Responsive Genes

**Figure 7:**
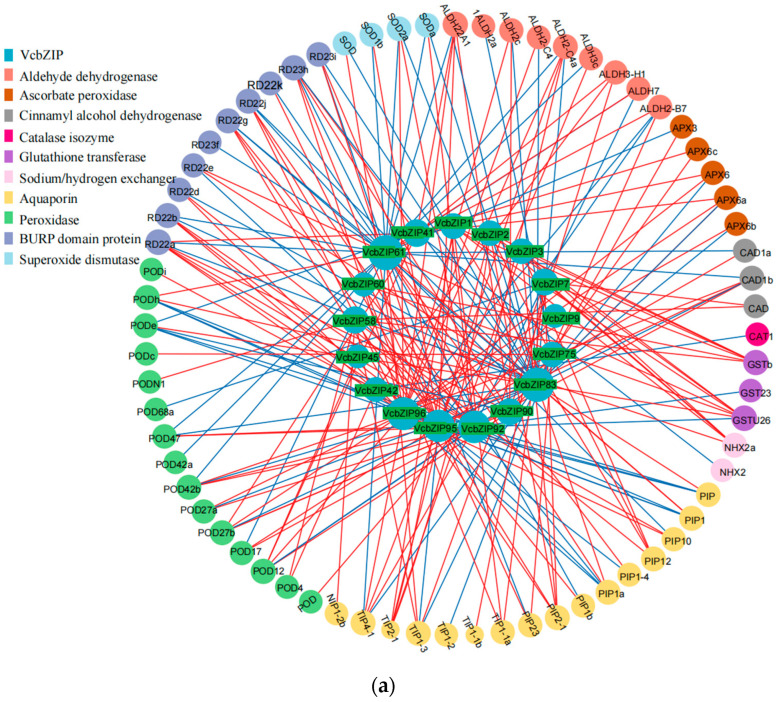

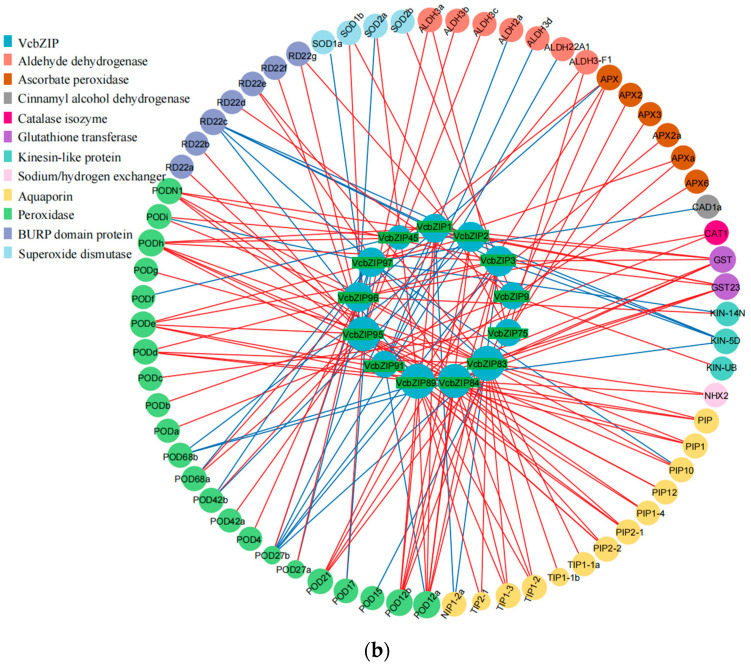
The network of Pearson correlation between *VcbZIP* genes and stress-responsive genes under salt (**a**) and drought (**b**) stress. Red lines indicate positive correlations; blue lines indicate negative correlations. The size of the circle represents the number of related genes.

We screened the aldehyde dehydrogenase (ALDH), ascorbate peroxidase (APX), cinnamyl alcohol dehydrogenase (CAD), CAT, GST, kinesin-like protein (KIN), sodium/hydrogen exchanger (NHX), aquaporin (AQP), peroxidase (POD), BURP domain protein (RD22), and SOD family genes (Tables S8 and S9) to further explore the roles of VcbZIPs in plant responses to salt and drought stress. The Pearson correlation coefficients (r) showed that *VcbZIPs* were significantly correlated with 86 stress-responsive genes in both salt and drought stress. Of these genes, *VcbZIP61* and *VcbZIP84* were significantly correlated with 34 and 32 stress-responsive genes under salt stress, respectively. However, *VcbZIP9* and *VcbZIP45* were significantly correlated with only POD genes under salt stress. *VcbZIP1*, *VcbZIP2*, and *VcbZIP3* were all positively correlated with *GSTs* (*GSTb* and *GSTU26*), *NHX2a*, and *AQPs* (*PIP12*, *PIP2-1*, and *TIP2-1*) under salt stress (Figure 7a). *VcbZIP83* and *VcbZIP89* were positively or negatively correlated with 22 stress-responsive genes, and *VcbZIP45* was positively correlated with only *PODh* genes under drought stress. We also found that *VcbZIP1*, *VcbZIP2*, and *VcbZIP3* were all positively correlated with *GSTs* (*GST* and *GST23*) and *PODs* (*POD12a*, *POD12b*, and *POD27b*) and negatively correlated with *KIN-5D*, *POD27b*, and RD22c under drought stress (Figure 7b).

## 3. Discussion

### 3.1. Structural Characteristics of the VcbZIP Family Genes

The number of genes in the *bZIP* gene family is large, and this family has been identified in various plant species. In the present study, we identified 102 *VcbZIP* genes through the reference genome of blueberry. This number of identified *VcbZIPs* is higher than those for Arabidopsis (78 bZIPs), rice (*Oryza sativa*) (89 *bZIPs*), grapevine (55 *bZIPs*) peach (47 *bZIPs*), and strawberry (50 bZIPs), and lower than those for wheat (*Triticum aestivum*) (227 *bZIPs*), soybean (*Glycine max*) (138), and apple (116) [1,8,9,10,41,42,43]. This difference may be related to differences in genome size or evolutionary histories between the species [39,44]. Using Arabidopsis as a taxonomic basis, the 102 VcbZIP TFs were divided into 10 groups. We found that VcbZIPs were distributed in 10 groups of Arabidopsis, and the same groups have similar gene structures in the different plant species (Figure 1). Group S represents small protein size, and group B represents large protein size in Arabidopsis [1]. Our study also found that the protein size of the VcbZIP proteins was small in group S and large in group B (Table S1; Figure 2). Group S comprises the largest bZIP cluster of 17 generally intron-less genes in Arabidopsis [1]. In our study, group S consists of 21 members and represents the second largest group, and most VcbZIP genes in this group showed the absence of introns. The same pattern has been found in many species, such as tea plant (*Camellia sinensis*), grapevine, apple, and rice [8,9,41,45]. Thus, bZIP proteins exhibited high conservation in the same group for different plant species, indicating that bZIP families in different species might share a common ancestor and similar evolutionary patterns to adapt to the environment. However, groups J, K, and M are lost in blueberry, implying that some *VcbZIP* genes might be lost or differentiated into other groups during the evolution process. Similar results have been seen in other plant species [46,47,48].

To further elucidate the structural characteristics of the blueberry VcbZIPs, we analyzed their conserved motifs, conserved domains, and exon–intron structures (Figure 2). Conserved motifs 1 and 2 were present in all groups and are related to bZIP domains. We also found that conserved motifs in the same phylogenetic group of VcbZIPs were similar. For example, most genes in group A contain conserved motifs 3 and 4, and tandem motif 7 was only present in group I. The VcbZIPs also contain various bZIP domains, such as bZIP_HY5-like domain in group H, bZIP_pant_BZIP46, and bZIP superfamily domains in group A; the bZIP_plant_GBF1 domain in groups C and S; bZIP_HBP1b-like domain in group D; and bZIP_plant_RF2 in groups E and I. Similar results have been observed in other species [49].

An analysis of exon–intron structures showed that *VcbZIP* contains 0 to 13 introns, with 15% of the genes containing no introns. The same pattern has been found in rice, apple, and tobacco, in which 19.1, 20.5%, and 16.88%, respectively, of such genes display an absence of introns [9,41,48]. Furthermore, the number of introns in the same group of *VcbZIP* genes is similar. For example, most *VcbZIP* genes in group S contained 0 introns, group I contained 3–4 introns, group A genes contained 0–5 introns in group, group C contained 4–5 introns, group D contained 10–11 introns, and *VcbZIP36–41* of group G contained 10–12 introns. Overall, the *VcbZIP* gene structures displayed diversity, and *VcbZIP* genes with close phylogenetic relationships may possess similar gene structures, among which the *VcbZIP* gene structures of groups G and D were more complex. In general, the rate of intron loss is faster than the rate of intron gain. Thus, groups G and D might contain the original genes. This gene structure feature of *VcbZIPs* has also been observed in grapevine and apple [8,9].

### 3.2. VcbZIPs Play Important Roles in Plant Responses to Salt and Drought Stress

**Figure 8:**
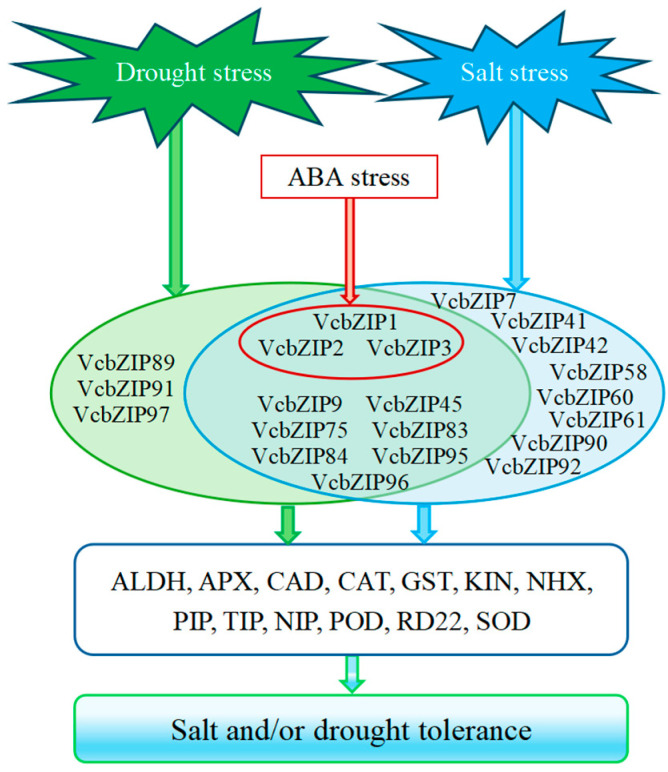
Model of VcbZIP regulation of salt and drought stress tolerance via the integration of salt, drought, and ABA stress. The green, blue, and red circles and arrows indicate the pathway of VcbZIPs in response to drought, salt and ABA stress, respectively.

The bZIP TFs participate in a broad range of cellular responses to biotic and abiotic stress, and their roles in providing stress tolerance in many plants have been reported. In the case of potato (*Solanum tuberosum*), 90 *StbZIPs* were identified, of which 13 *StbZIP* genes were differentially expressed under salt stress, according to RNA-seq data [50]. Regarding the tea plant, 29 *TEACsbZIP* genes showed upregulated expression, while three genes were downregulated in drought treatment [45]. In the case of grapevine, RT-PCR analysis of the *VvbZIP* gene expression showed that 25 *VvbZIP* genes were upregulated, and 7 *VvbZIP* genes were downregulated under drought treatment, and most differentially expressed *VvbZIP* genes belonged to groups A and S of Arabidopsis [8]. In our study, 18 and 13 *VcbZIPs* were differentially expressed in response to salt and drought stress, respectively; thus, these genes may be involved in salt and drought tolerance (Figure 8). The most differentially expressed *VcbZIP* genes were identified in group S (*VcbZIP75*, *VcbZIP83*, *VcbZIP84*, *VcbZIP89*, *VcbZIP90*, *VcbZIP91*, and *VcbZIP92*), group A (*VcbZIP1*, *VcbZIP2*, *VcbZIP3*, *VcbZIP7*, and *VcbZIP9*), and group F (*VcbZIP95*, *VcbZIP96*, and *VcbZIP97*). In Arabidopsis, the genes from groups A, S, and F are also mainly involved in abiotic stress [13,18,19]. Interestingly, most of the differentially expressed *VcbZIP* genes, including *VcbZIP1*, *VcbZIP2*, *VcbZIP3*, *VcbZIP9*, *VcbZIP45*, *VcbZIP75*, *VcbZIP83*, *VcbZIP84*, *VcbZIP95*, and *VcbZIP96,* were all induced by salt and drought stress. *VcbZIP1*, *VcbZIP2*, *VcbZIP3*, *VcbZIP83*, *VcbZIP84*, and *VcbZIP75* were significantly upregulated or downregulated by more than four times under salt or drought stress compared to the 0 h control, implying that these genes may be responsible for blueberry leaf salt or drought tolerance.

### 3.3. Functional Prediction of VcbZIPs Under Salt and Drought Stress

In Arabidopsis, group A consists of 13 members and can be further classified into four distinct subgroups. The ABF subgroup contains the highly related ABF1–4, which have been found to act at the core of ABA signaling in a partially redundant manner [1,12]. In our study, VcbZIP1–7 were clustered in this subgroup, and functional annotation showed that these genes belong to ABFs (Figure 1; Table S1). Most studies have shown that ABFs can be transcriptionally induced by abiotic stress that affects water supply, such as salinity and drought, and post-transcriptionally activated by ABA signaling, thus achieving adaptive responses to counteract water deficit in plant tissues [5,51]. Arabidopsis *ABF1–4* are highly induced by salt and drought stress and ABA in vegetative tissues [52,53,54,55]. ABF2, ABF3, and ABF4 confer drought and/or salt tolerance to plants through their involvement in ABA signaling and are master transcription factors in ABA signaling that play cooperative roles in salt and drought stress tolerance [13,53,56,57,58,59]. In rapeseed (*Brassica napus* L.), the *BnaABF2* gene enhances salt and drought tolerance in transgenic Arabidopsis [60]. In cotton (*Gossypium hirsutum*), overexpression of *GhABF3* increases tolerance to salt and drought [32]. In this study, we found that *VcbZIP1*, *VcbZIP2*, and *VcbZIP3* (homolog *AtABF2*) were upregulated by salt and drought, and *VcbZIP7* (homolog *AtABF2*) was induced by salt stress. There is also a significant correlation between *VcbZIP1*, *VcbZIP2*, and *VcbZIP3* under both salt and drought stress, and these genes also were upregulated by ABA stress, implying that VcbZIP1, VcbZIP2, and VcbZIP3 may cooperatively mediate stress-responsive via ABA signaling (Figure 8).

Besides the ABF subgroup, the DPBF subgroup contained DPBF1/ABI5, DPBF2, DPBF3/AREB3, and DPBF4/EEL, which have also been found to be central in ABA signaling. DPBF1/ABI5 has been extensively characterized with respect to its function in ABA-dependent seed maturation and seed germination and has been found to directly control genes involved in abiotic stress responses [61]. For example, Arabidopsis actin-depolymerizing factor (ADF5) increases tolerance to drought stress, and DPBF3 can bind to the *ADF5* promoter and activate its transcription [62]. Barley HvABI5 is involved in ABA-dependent drought stress [63]. In our study, *VcbZIP9* was upregulated by salt and drought stress and clustered with *DPBF3* (At3G56850) and *DPBF4* (At2G41070) together. However, exogenous ABA did not induce VcbZIP9 expression. Thus, whether blueberry responds to abiotic stress through the ABA pathway requires further experimental evidence.

In addition to the *VcbZIPs* of group A, *VcbZIP75*, *VcbZIP83*, and *VcbZIP84* of group S, *VcbZIP95* and *VcbZIP96* of group F, and *VcbZIP45* of group H were also induced by salt and drought stress. *VcbZIP89* and *VcbZIP91* of group S and *VcbZIP97* of group F were induced in response to drought stress, and *VcbZIP90* and *VcbZIP91* (group S); *VcbZIP58*, *VcbZIP60*, and *VcbZIP61* (group I); *VcbZIP41* (group G); and *VcbZIP42* (group E) were induced in response to salt stress according to RNA-seq data. Several studies showed that Arabidopsis bZIPs from groups S, I, and C also involved various stresses. For example, Arabidopsis *AtbZIP11* and *AtbZIP53* in group S were responsive to high salt stress [64]. Rice *OsbZIP16* (Arabidopsis group S) positively regulates drought tolerance, and overexpression of potato *StbZIP-65* (group S) in Arabidopsis enhances salt tolerance [65,66]. Cotton bZIP TF GhVIP1 (Arabidopsis group I) can enhance drought tolerance [67]. However, most studies have shown that group S1 bZIPs (AtbZIP1, AtbZIP2, AtbZIP11, AtbZIP44, and AtbZIP53) preferentially form heterodimers with group C bZIPs (AtbZIP9, AtbZIP10, AtbZIP25, and AtbZIP63) to regulate metabolic reprogramming during stress [68,69,70,71]. The apple C/S1 bZIP network (MdbZIP2 and MdbZIP39 from group S and MdbZIP80 from group C) directly suppressed the expression of MdIPT5b, thus negatively modulating drought tolerance in a functionally redundant manner [72]. In this study, six *VcbZIPs* (including *VcbZIP83*, *VcbZIP84*, and *VcbZIP89–92*) belonging to group S1 were upregulated or downregulated under drought or salt stress. However, group C members were not induced by salt or drought stress. Then, we calculated Pearson correlation coefficients between the group S1 members and other differentially expressed *VcbZIPs* and found that *VcbZIP84* was significantly correlated with some members from groups I, S, and F; *VcbZIP84* with members from groups G and E; and *VcbZIP92* with *VcbZIPs* from groups S, F, and I during salt stress. *VcbZIP83*, *VcbZIP84*, and *VcbZIP89* were significantly correlated with *VcbZIPs* from groups S, A, or F in the case of drought. Thus, these VcbZIP proteins may regulate stress tolerance in a functionally redundant manner or in a regulatory network.

In Arabidopsis, group H consists of the HY5 and HY5 homolog (HYH) [1]. HY5 is a master regulator that integrates multiple signaling pathways to coordinate plant tolerance to diverse stresses, including light, hormones, and low and high temperatures [73,74,75,76,77]. Previous studies have shown that *VcHY5* was induced by UV-B radiation in blueberry callus [78]. In this study, we found that *VcHY5* (*VcbZIP45*) was responsive to both salt and drought stress. However, *VcHY5* was downregulated by salt and upregulated by drought. Thus, VcHY5 may be involved in multiple stress-responsive signaling pathways and play different roles in various stresses.

### 3.4. VcbZIPs May Modulate Salt and Drought Tolerance by Regulating the Expression of Stress-Responsive Genes

bZIP proteins regulate gene expression by interacting with ACGT elements in the promoter regions of downstream genes and sequences flanking the ACGT core, affecting bZIP protein-binding specificity [79,80,81]. Some studies showed that overexpression of bZIP enhanced salt or drought tolerance by altering the expression of stress-regulated genes [13,58,59]. In this study, we screened the stress-responsive genes, of which the ALDH, APX, CAD, CAT, GST, KIN, NHX, AQP, POD, RD22, and SOD family genes were positively and negatively correlated with VcbZIP genes during salt or drought stress. Thus, VcbZIP proteins may regulate salt or drought stress by affecting the expression of these stress-responsive genes (Figure 8).

## 4. Materials and Methods

### 4.1. Identification of VcbZIP Genes in the Blueberry Genome

To identify putative VcbZIPs, the hidden Markov model (HMM) profile of the bZIP_1 (PF00170), bZIP_2 (PF07716), bZIP_Maf (PF03131), and bZIP_C (PF12498) domains was downloaded from the Pfam Protein Family database (http://pfam.xfam.org, accessed on 20 June 2024), and sequences of *bZIP* genes from Arabidopsis were downloaded from the Arabidopsis Information Resource database (TAIR, https://www.arabidopsis.org/, accessed on 20 June 2024). The four bZIP domains and *bZIP* genes of Arabidopsis were used as a query against the Genome Database for *Vaccinium corymbosum* cv. Draper V1.0 genome sequence, with a threshold E-value of ≤1 × 10^−5^ (GDV, https://www.vaccinium.org/, accessed on 25 June 2024). The candidate bZIP proteins of blueberry were searched in the SMART (http://smart.embl-heidelberg.de/, accessed on 28 June 2024) and CDD (https://www.ncbi.nlm.nih.gov/Structure/cdd/wrpsb.cgi, accessed on 29 June 2024) databases, and genes without bZIP domains in their encoded proteins were removed. After the repeat and incomplete sequences were manually removed, 102 *VcbZIP* genes encoding complete bZIP domains were obtained and were named from *VcbZIP1* to *VcbZIP102* based on phylogenetic trees. The online program ExPASy (https://web.expasy.org/protparam/, accessed on 10 July 2024) was used to calculate the instability index (Ii), aliphatic index (AI), and grand average of hydropathicity (GRAVY) of the VcbZIP proteins, and the online program WoLF PSORT (https://www.genscript.com/wolf-psort.html?src=leftbar, accessed on 12 July 2024) was used to predict their subcellular localizations.

### 4.2. Phylogenetic Analysis

The 78 bZIP amino acid sequences of A. thaliana were downloaded from NCBI (https://www.ncbi.nlm.nih.gov/, accessed on 20 July 2024) and used as references to categorize the VcbZIP proteins. The phylogenetic trees were constructed in MEGA X using the unweighted pair group method and the arithmetic average (UPGMA) method. The phylogenetic trees were divided into different groups, referring to studies on Arabidopsis [1].

### 4.3. Analysis of Conserved Motifs, Conserved Domains, and Gene Structures

The deduced amino acid sequences of the VcbZIP were uploaded to the online search tool MEME 4.10.1 (http://meme-suite.org/tools/meme, accessed on 10 August 2024) for conserved motif analysis, with the maximum number of motifs being set at 10 and the order of site distribution set to zero or one occurrence per sequence. The conserved domains of VcbZIP proteins used for visualization were predicted using the online program CDD (https://www.ncbi.nlm.nih.gov/Structure/bwrpsb/bwrpsb.cgi, accessed on 10 August 2024). Generic feature format files of the VcbZIP family members were downloaded from the Genome Database for *Vaccinium*, including sequence information for the untranslated regions (UTRs), exons, and introns. TBtools-II software (version 2.003) was used to visualize the results of the conserved motifs, conserved domains, and gene structure [82].

### 4.4. Analysis of Cis-Acting Regulatory Elements in the Promoters of VcbZIP Genes

The upstream 2000 bp sequence of each *VcbZIP* gene was downloaded from the Genome Database for *Vaccinium*. The putative cis-regulatory elements related to phytohormone and abiotic stress responses were predicted using the online program PlantCARE (https://bioinformatics.psb.ugent.be/webtools/plantcare/html/, accessed on 20 August 2024) and mapped along the presumptive promoters using TBtools-II software (version 2.152).

### 4.5. Differentially Expressed VcbZIP Genes Under Drought, Salt, and ABA Stress

The RNA-seq data from the blueberry cultivar Northland under drought (0, 6, 12, 24, or 48 h), salt (0, 6, 12, 24, or 48 h), and ABA (0, 6, or 12 h) stress were downloaded from the BioProject database in the NCBI repository (https://www.ncbi.nlm.nih.gov/bioproject; accession numbers PRJNA1160404, PRJNA1128395, and PRJNA997066, respectively, accessed on 10 September 2024) [83,84,85]. Gene expression levels were estimated based on fragments per kilobase of transcript per million fragments mapped (FPKM). Differentially expressed genes between stress-treated samples and the control (0 h) sample were identified based on an absolute log2-fold change (FC) value of ≥1 and a *p* value of <0.05 using DESeq2 (http://bioconductor.org/packages/release/bioc/html/DESeq2.html, accessed on 20 September 2024). The heatmaps of differentially expressed *VcbZIP* genes based on log_10_ (FPKM) values under drought (0, 6, 12, 24, or 48 h) and salt (0, 6, 12, 24, or 48 h) stress were constructed with TBtools-II software (version 2.152). The functions of differentially expressed *VcbZIP* genes were annotated using the Kyoto Encyclopedia of Genes and Genomes (KEGG), Eukaryotic Orthologous Groups (KOG), Protein Family (Pfam), SwissProt (a manually annotated and reviewed protein sequence database), and NCBI non-redundant protein sequences (NR) databases [86,87,88,89,90]. The differentially expressed stress-responsive genes from aldehyde dehydrogenase (ALDH), ascorbate peroxidase (APX), cinnamyl alcohol dehydrogenase (CAD), catalase isozyme (CAT), glutathione S-transferase (GST), kinesin-like protein (KIN), sodium/hydrogen exchanger (NHX), aquaporin (AQP), peroxidase (POD), BURP domain protein (RD22), and superoxide dismutase (SOD) families were screened under drought or salt stress. Pearson correlation coefficients (r) were calculated to determine the correlation between genes under drought or salt stress according to the FPKM values using SPSS 19.0 software (IBM, Armonk, NY, USA).

### 4.6. Plant Materials and Stress Treatment

The plants of the blueberry (*Vaccinium corymbosum*) cultivar Northland were subjected to salt and drought treatment. The six-month-old blueberry plants were grown in a growth chamber at 25 °C with 70% relative humidity with a 16 h light/8 h dark photoperiod at Jilin University, China. The potted plants were irrigated with 1/2 Hoagland solution containing 200 mM NaCl for salt stress or 20% polyethylene glycol 6000 (PEG 6000) for drought stress [91,92,93]. The first to third fully expanded leaves were collected from randomly selected branches after 6 h (S6), 12 h (S12), 24 h (S24), and 48 h (S48) of NaCl treatment and 6 h (P6), 12 h (P12), 24 h (P24), and 48 h (P48) of PEG 6000 using samples collected at time 0 h (S0/P0) with 1/2 Hoagland solution (without NaCl and PEG 6000) as a control. About 100 branches were sampled for each treatment. The leaves were flash-frozen in liquid nitrogen and stored at −80 °C for RNA extraction [83,84].

The total RNA was extracted using a Plant Total RAN Isolation Kit (Sangon Biotech, Shanghai, China), and first-strand cDNAs were synthesized using PrimeScript RT Master Mix (Takara Bio, Kusatsu, Japan). The differentially expressed VcbZIP genes under salt and drought stress were subjected to RT-qPCR analysis using an ABI 7900HT real-time PCR system (Thermo Fisher Scientific, Waltham, MA, USA) and were used to validate the accuracy and reliability of RNA-seq data. Glyceraldehyde-3-phosphate dehydrogenase (GAPDH; GenBank accession no. AY123769) was used as the reference gene. The primer sequences of VcbZIP genes are shown in Table S10, and the relative expression levels of VcbZIP genes were calculated using the 2^−∆∆Ct^ method. All experiments were carried out with three independent biological replicates, and three technical replicates were performed for each biological replicate. Tukey’s test was used to identify significant differences at a *p* value of ≤0.05 using SPSS 19.0 software.

## 5. Conclusions

In conclusion, a total of 102 putative VcbZIP genes were identified and classified into 10 groups. The motifs, domains, and exon–intron structures among the same phylogenetic groups of VcbZIPs were often highly conservative. RNA-seq data showed that 18 *VcbZIPs* responded to salt stress, and 13 responded to drought stress. Of these genes, *VcbZIP1–3* were induced not only by salt and drought stress but also by ABA stress, indicating that VcbZIP1–3 might act as bridges integrating ABA signaling and salt and drought stress signaling. Furthermore, we screened *VcbZIP1*, *VcbZIP2*, *VcbZIP3*, *VcbZIP83*, *VcbZIP84*, and *VcbZIP75* as candidate genes to improve salt or drought tolerance via genetic engineering. The VcbZIP transcription factors regulated stress-responsive genes to improve drought or salt tolerance. Our findings provide a useful reference for subsequent research investigating the biological function of VcbZIP family members to guide breeding efforts for improved abiotic stress tolerance in blueberry.

## Data Availability

All data in this study are included in the Supplementary Materials.

## Supplementary Materials

The following supporting information can be downloaded at https://www.mdpi.com/article/10.3390/ijms26020843/s1.
