# Supplementary material for: Genome-Wide Analysis of bZIP Transcription Factors and Expression Patterns in Response to Salt and Drought Stress in Vaccinium corymbosum
Source: Int J Mol Sci. 2025 Jan 20;26(2):843. doi: 10.3390/ijms26020843 (PMC11766362; doi:10.3390/ijms26020843)
Supplement: Supplementary file 1 [file ijms-26-00843-s001.zip › Figure S1.pdf]

Motif 1: RRQKRMJSNRESAARSRERKQAY

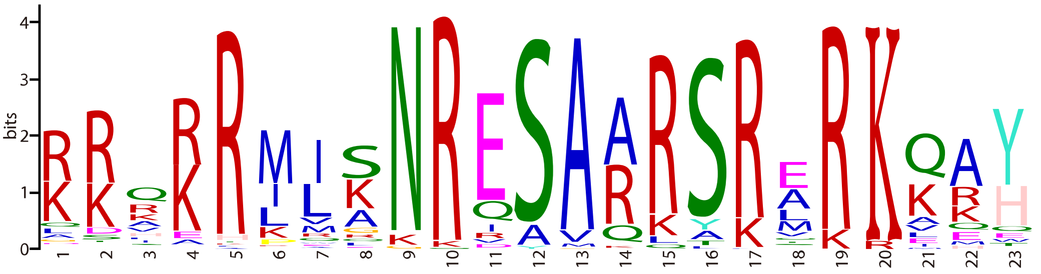

Motif 2: ELEREVAQLREENAELKKRLQ

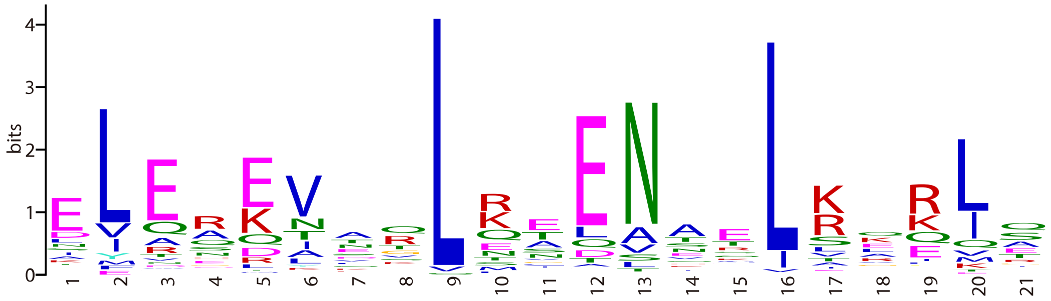

Motif 3: SFGSMTLDEFL

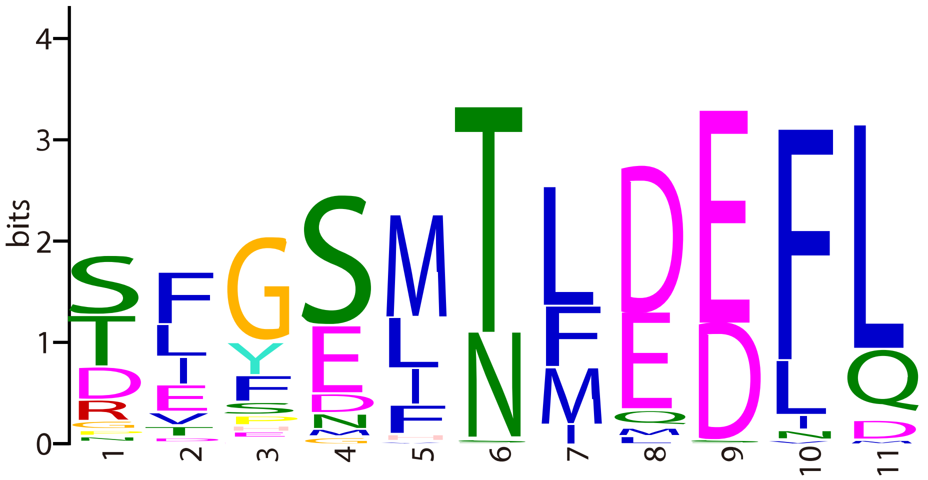

Motif 4: SAGQDESVAPSSSLQRQGSLTLPRTLSQKTVDEVWREJIKGQGGGQNPGT

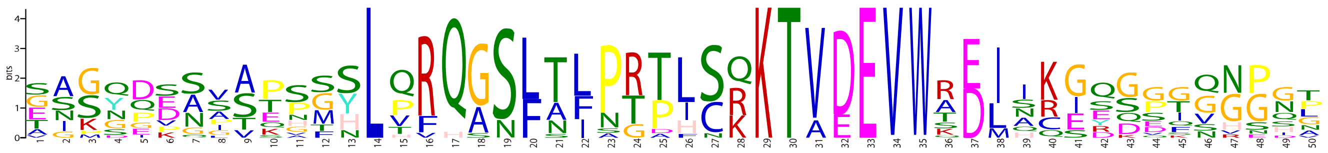

Motif 5: AMEQQAKLRDALNEALKQEVERLKIATGE

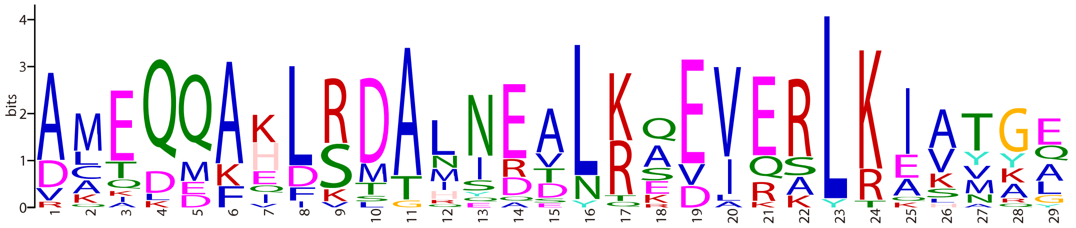

Motif 6: PPGGTPPHTYTPMHPHGFIYS

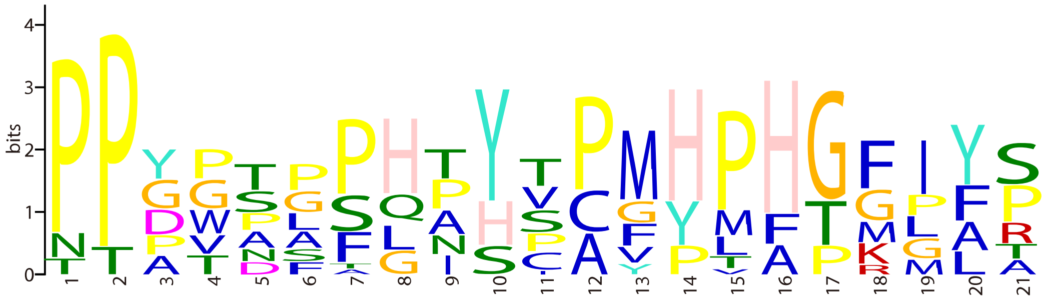

Motif 7: PTVYYSPLTSPSTVQ

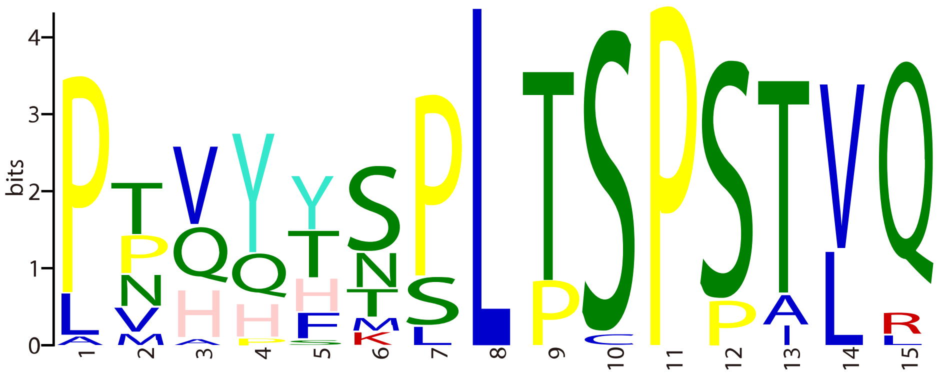

Motif 8: GVPGPTTNLNIGMDYWGAAINGPLHSLNGBAPHSPVAGGIV

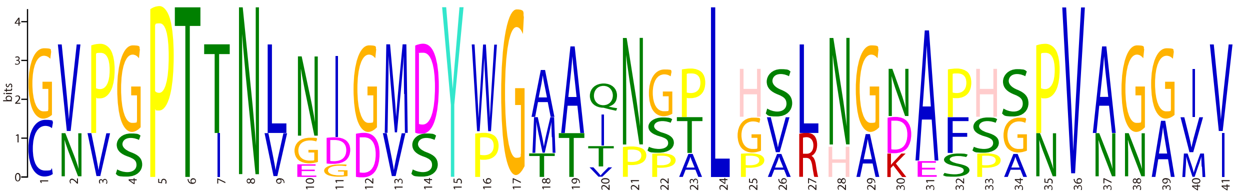

Motif 9: FSPPSPNGPAEASGNTNSSVETDGKLEEGKEKLPJKRSKAS

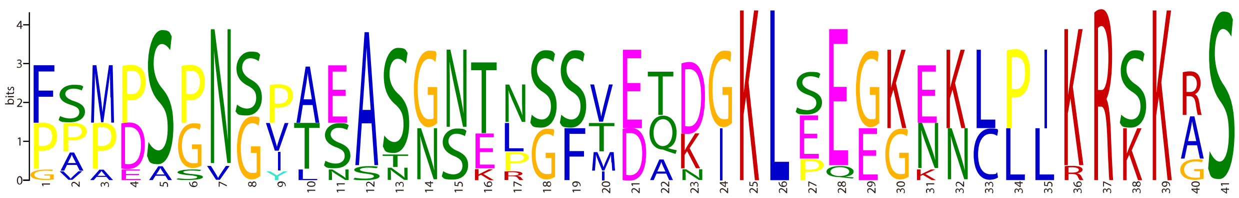

Motif 10: SSESKKAMAPDKLAEJWLEDP

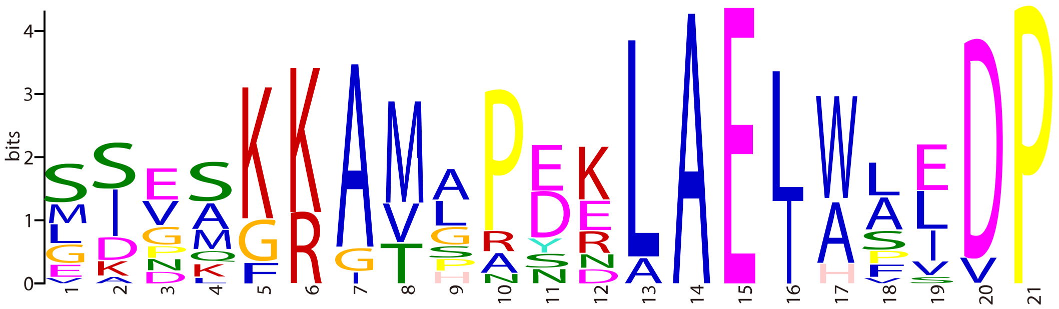

Figure S1 The 10 conserved motifs were predicted using the MEME website and the letter height of the amino acid residue represents its conservation degree.
